# Community health workers impact on maternal and child health outcomes in rural South Africa – a non-randomized two-group comparison study

**DOI:** 10.1186/s12889-020-09468-w

**Published:** 2020-09-17

**Authors:** Karl W. le Roux, Ellen Almirol, Panteha Hayati Rezvan, Ingrid M. le Roux, Nokwanele Mbewu, Elaine Dippenaar, Linnea Stansert-Katzen, Venetia Baker, Mark Tomlinson, M. J. Rotheram-Borus

**Affiliations:** 1 Institute for Life Course Health Research, Department of Global Health, Stellenbosch University, Tygerberg, South Africa; 2 Zithulele Training and Research Centre, Zithulele Hospital, Ginyintsimbi Village, Eastern Cape South Africa; 3 Family Medicine Department, Walter Sisulu University, Mthatha, South Africa; 4 Primary Healthcare Directorate, University of Cape Town, Cape Town, South Africa; 5 Department of Psychiatry and Biobehavioral Sciences, Semel Institute, University of California, 10920 Wilshire Boulevard, Suite 350, Los Angeles, CA 90024-6521 USA; 6 Philani Maternal, Child Health and Nutrition Trust, Site C, Khayelitsha, Cape Town, South Africa; 7 School of Nursing and Midwifery, Queens University, Belfast, UK

**Keywords:** Community health workers, Rural, Eastern cape, South Africa, Wasting, Depression

## Abstract

**Background:**

Home visits by paraprofessional community health workers (CHWs) has been shown to improve maternal and child health outcomes in research studies in many countries. Yet, when these are scaled or replicated, efficacy disappears. An effective CHW home visiting program in peri-urban Cape Town found maternal and child health benefits over the 5 years point but this study examines if these benefits occur in deeply rural communities.

**Methods:**

A non-randomized, two-group comparison study evaluated the impact of CHW in the rural Eastern Cape from August 2014 to May 2017, with 1310 mother-infant pairs recruited in pregnancy and 89% were reassessed at 6 months post-birth.

**Results:**

Home visiting had limited, but important effects on child health, maternal wellbeing and health behaviors. Mothers reported fewer depressive symptoms, attended more antenatal visits and had better baby-feeding practices. Intervention mothers were significantly more likely to exclusively breastfeed for 6 months (OR: 1.8; 95% CI: 1.1, 2.9), had lower odds of mixing formula with baby porridge (regarded as detrimental) (OR: 0.4; 95% CI: 0.2, 0.8) and were less likely to consult traditional healers. Mothers living with HIV were more adherent with co-trimoxazole prophylaxis (*p* < 0.01). Intervention-group children were significantly less likely to be wasted (OR: 0.5; 95% CI 0.3–0.9) and had significantly fewer symptoms of common childhood illnesses in the preceding two weeks (OR: 0.8; 95% CI: 0.7,0.9).

**Conclusion:**

The impact of CHWs in a rural area was less pronounced than in peri-urban areas. CHWs are likely to need enhanced support and supervision in the challenging rural context.

## Background

Rural populations face greater challenges than their urban counterparts in accessing good quality health care. Not only are health facilities in rural areas less accessible due to distance and topography, but clinics and hospitals are often under-resourced, poorly maintained [1, 2] and lack essential medicines [3]. Furthermore, there may be a scarcity of trained and skilled healthcare workers in rural areas, including doctors, pharmacists and nurses leading to suboptimal health outcomes [2, 4].

One proposed remedy to the shortage of human resources is training and employing lay healthcare workers, usually known as village or community healthcare workers (CHWs). CHWs are typically community members who are trusted and respected, and able to provide a link between people’s homes and formal government primary health care (PHC) clinics [5]. The efficacy of CHWs in reducing the burden of care in understaffed and under-resourced health systems remains a point of debate, with their perceived value varying significantly [6, 7].

In 2011, the South African (SA) National Department of Health (NDOH) launched ‘The Re-engineering of Primary Health Care’ policy, which relies heavily on CHWs, to reduce maternal and child mortality and improve access to health care [8–11]. Successful CHW programs have provided services to over 200 million people over the past 2 decades in Brazil, Bangladesh and Nepal [12]. CHWs have implemented preventive interventions for maternal and child health [13–15]. CHW programs have been shown to reduce child mortality [16, 17], reduce maternal depression [18, 19], improve access to health care [7] and improve child growth and development [18–20]. Despite good evidence that well-managed CHW programs can positively influence a range of health outcomes, programs often face a myriad of practical obstacles that impact their effectiveness, particularly when expanded and replicated, and most CHW programs have not been able to be taken to scale whilst retaining effectiveness [21].

There has, therefore, been a call for more research on how to best implement CHW programs. This study builds on a series of evaluations of the Philani CHW program in Khayelitsha, Cape Town, which has shown long term benefits for maternal and child health outcomes [18, 19, 22–24]. This paper evaluates whether the CHW home-visiting model is equally effective in a deeply rural area where health providers and patients face many additional challenges in seeking and consistently receiving care.

## Methods

### Setting

The study was initiated in the rural Eastern Cape, in the catchment area of four clinics referring to Zithulele District Hospital, situated in the King Sabata Dalidyebo Subdistrict of the OR Tambo district, one of the poorest municipalities in South Africa [25]. The catchment population is approximately 40,000, with a density of (124 people/km^2^) [26] and the topography is hilly with deep river valleys and gorges. This, combined with poor road infrastructure, makes access to healthcare services challenging for much of the population.

### Study design

**Fig. 1:**
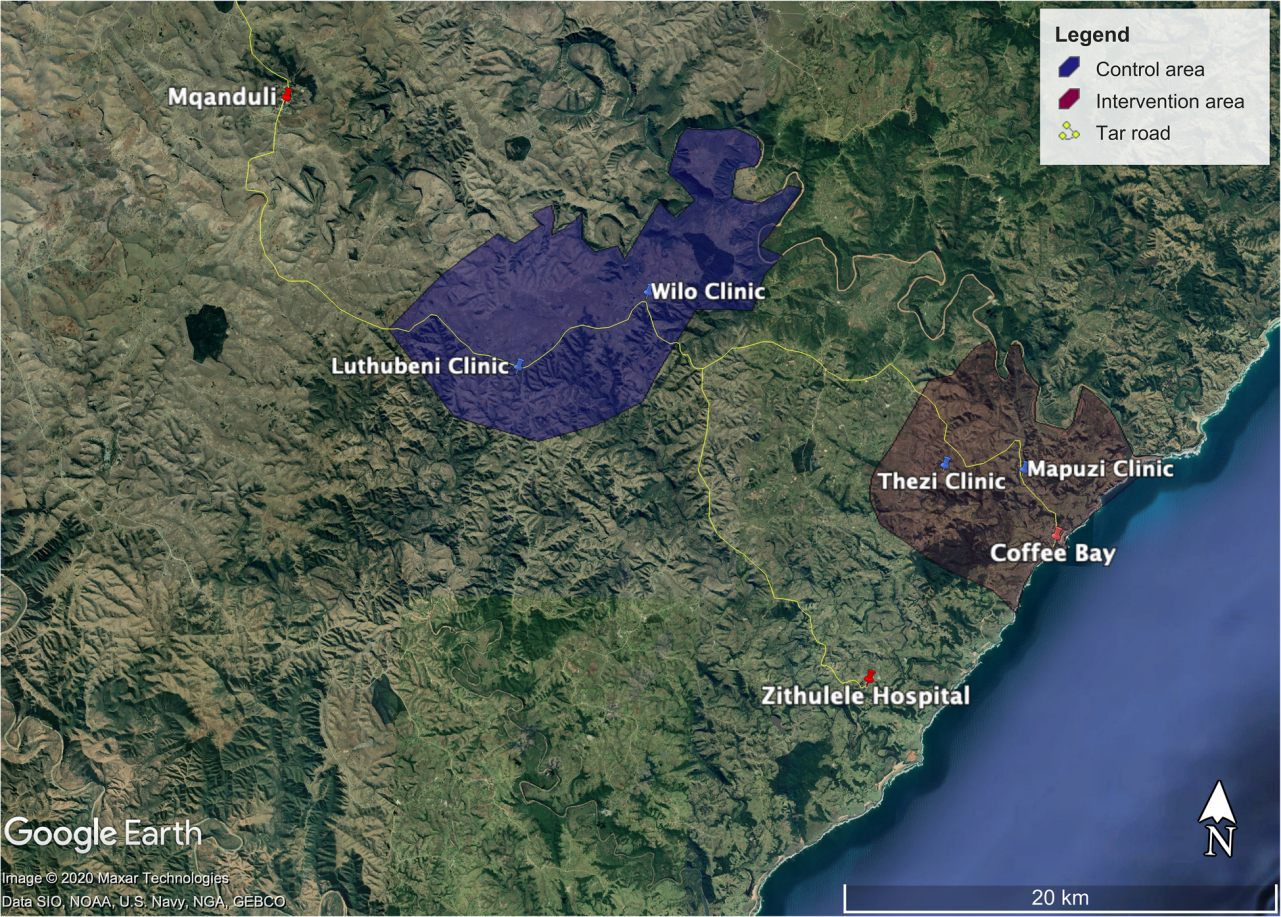
Control and intervention areas, Eastern Cape, South Africa

This study was an early Phase II non-randomized two-group comparison study evaluating the Philani Home-Visiting Model in the rural Eastern Cape of South Africa, called the Zithulele Mothers-to-be Assessment (ZiMBA). Recruitment started in August 2014 and ended in May 2017. Mothers were assigned based on the location of clinic: (1) Mapuzi and Tshezi, an area with home visiting by CHWs, which we will refer to as the “intervention” group or (2) Wilo and Luthubeni, areas with standard clinical care and no CHWs, as the “comparison” group. Philani CHWs focusing on maternal and child health, had been assigned to conduct home visits within designated geographical areas (i.e. intervention areas a year previously). The comparison area was matched with the intervention area based on clinic coverage, size, and distance from the district hospital by tar road, and a similar number of expected pregnancies, which was estimated from data of the Zithulele Births Follow-up Study (ZiBFUS) [27]. We ensured that a buffer zone existed between the two areas to avoid “cross-contamination” of intervention effects. See map – Fig. 1.

#### Intervention group

Philani Maternal, Child Health and Nutrition Trust is a Non-Governmental Organization (NGO) that has been operating in peri-urban townships of Cape Town since 1979. Philani Mentor Mothers (Community Health Workers) are positive-deviant women whose children are thriving despite living in poverty [9]. These mothers are recruited to work in the areas where they live and are primarily trained to address maternal and child health issues in their communities at the level of the household. They perform regular house-to-house visits in their designated neighborhoods and identify both pregnant mothers and malnourished children who are then offered to entry into the program and followed up with regular home visits. The Community Health Workers (CHWs) build strong relationships with their clients and encourage mothers to attend antenatal care, immunize their children, weigh them regularly and breastfeed exclusively for 6 months; they advise mothers about health-care regimes, mental-health issues, antenatal care, optimal infant feeding, accessing grants and accessing prevention of mother-to-child HIV transmission (PMTCT) [22]. CHW are also trained to identify and refer household members with possible TB and to support people living with HIV. A critical aspect of the intervention is the peer support provided to mothers who are struggling by women from their own community, who have faced similar challenges.

In 2010, the program was expanded to a deeply rural area around Zithulele District Hospital, and by 2013 to the Coffee Bay area - both in the OR Tambo district of the Eastern Cape. The CHW program was integrated into primary health-care services in the King Sabata Dalindyebo sub-district authority, and provides several advantages over other CHW programs, including a strong emphasis on care in households and the community rather than at primary care clinics, a total 6-week standardized training both in the classroom and the field, meticulous record-keeping of patient follow-ups, daily in-the-field support and supervision visits by supervisors ensuring accountability and the recruitment of positive-deviant mothers [9].

#### Comparison group

The Philani Mentor Mother program is not active in this area. Mothers and children in the comparison group have access to free primary health care at clinics and free maternity and child healthcare (up to 6 years) at government hospitals, which includes HIV care.

### Sample

All pregnant women attending antenatal care during the study period and who agreed to participate were recruited by trained interviewers stationed at each of the four clinics listed above, during the recruitment period. Women living in the areas covered by the four clinics who presented to Zithulele Hospital around the time of delivery, but who were not previously recruited, were also recruited into the study. Participants who were deaf, mute or with significant psychiatric issues at initial contact were excluded. All women signed an informed consent form. In addition, those under the age of 18 had a parent or guardian sign consent.

**Fig. 2:**
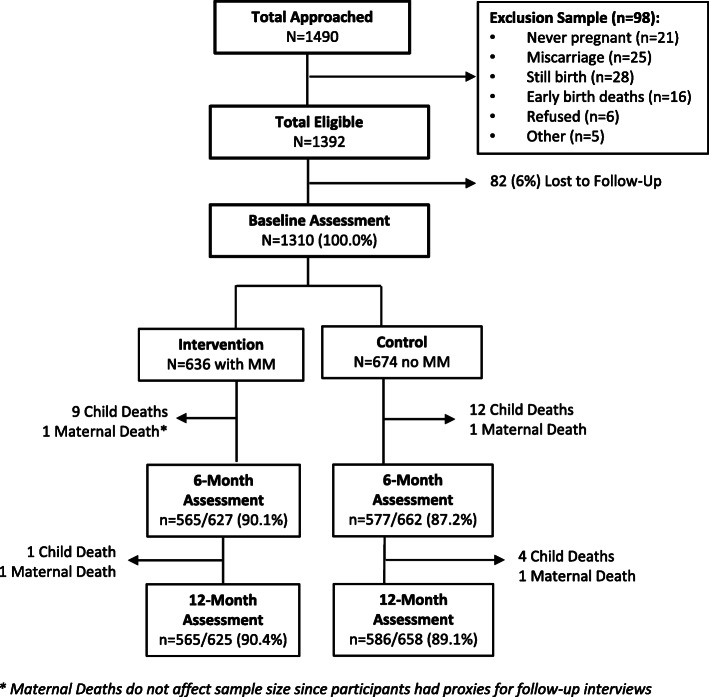
Participant flow chart - by intervention (Thezi-Mapuzi) and comparison (Luthubeni-Wilo) groups

Figure 2 depicts the participant flow chart. Of the 1490 mothers approached at antenatal clinics and the hospital, 180 women were not eligible due: no pregnancy (*n* = 21), miscarriages and intrauterine deaths (*n* = 53), early birth deaths (*n* = 13), refusal at baseline interview or other reasons (*n* = 11), and not completing the baseline interview (*n* = 82). A total of 1310 women were recruited into the study, stratified by location of clinic: (1) Mapuzi and Thezi (Intervention group; *n* = 636); or (2) Wilo and Luthubeni (Comparison group; *n* = 674). Baseline assessments were performed soon after the birth of the baby (median, 2 days) and at 6-months post-birth (median, 180 days). In the analysis, babies who died by 6-months (*n* = 21), twins or triplets (*n* = 17), and HIV-seropositive children (*n* = 3) were excluded. There were two maternal deaths by 6 months. Although child deaths did affect the overall study sample, maternal deaths did not, as long as there was a proxy for the mother’s interview (i.e. caregiver like a grandmother) who could complete the assessment.

### Measures

#### Demographic characteristics

Background characteristics collected included maternal age, highest education level achieved (years), current employment or learner status, and the presence of a live-in partner or husband. Economic resources were identified as monthly income (> 2000 South African Rand [ZAR]), receipt of the child support grant, access to electricity and safe water, and the number of adults and children that live in the household.

#### Alcohol use

*M*others were asked if they ever used alcohol before pregnancy, and if they continued to use alcohol after discovering they were pregnant.

#### Maternal health

Maternal antenatal cards detailed the medical care the mother received before pregnancy and the number of antenatal visits she attended. We collected previous pregnancy history including poor outcomes and the number of live births.

#### Maternal mental health

Depressive symptoms were measured using the Edinburgh Postnatal Depression Scale (EPDS). The EPDS is a 10-item scale with four Likert-type responses for each item, with mothers self-reports indicating possible depressed mood with scores > 13 and probable clinical depression ≥18 that has been extensively used in South Africa [24, 28–30] and has been found to “reliably and validly measure perinatal depression symptom severity or screen for probable postnatal depression in African countries” (29, p1).

#### HIV status and adherence to PMTCT

HIV testing during pregnancy was confirmed either by self-report or by the mother’s antenatal card at baseline. Mother’s HIV status, receipt of ARVs before and during pregnancy, and disclosure of HIV status were also recorded.

#### Paternal characteristics

At baseline, data collectors record if the child lives with the father, and the father’s HIV status and substance use.

#### Breastfeeding

current breastfeeding at the birth interview was recorded and how soon the infant was put to the breast. At the 6-months assessment, mothers self-reported whether they were exclusively breastfeeding for three and 6 months. If a mother was not exclusively breastfeeding, data collectors asked whether and when the mother started using formula, and whether she was mixing formula with baby porridge.

### Child outcomes

#### Birth outcomes

Birthplace of the child was either at the hospital, clinic, or on the way to the hospital/clinic versus at home. Low birth-weight was defined as any infant that weighed less than 2500 g at the time of birth. Finally, Apgar scores were recorded at birth at 5 min, ranging from 1 to 10 using information from the government Road to Health Card (RtHC), which is a health record kept by the mother.

#### Growth measures

Trained and certified interviewers weighed children (kilograms) and measured their length (centimeters) using a measuring mat at birth and 6-months. Birth weight (g/kg) was also recorded from the RtHC. Infant anthropometric data was then converted to z-scores based on the World Health Organization’s (W.H.O) age-adjusted norms [31]. A z-score below − 2 standard deviation (SD) was considered a serious growth deficit: <− 2 for height-for-age z-scores (HAZ) was considered stunted, < − 2 for weight-for-age z scores (WAZ) was considered underweight, and < − 2 for weight-for-height z scores (WHZ) were considered wasted. Growth scores of > 5 SD or < − 5 SD may have represented measurement errors and were replaced by 5 or − 5 in order to reduce the effect of outliers in our analyses (i.e. HAZ [*n* = 21, 0.6%], WAZ [*n* = 20, 0.6%], and WHZ [*n* = 123, 3.5%]).

#### Immunizations and vitamin a

Data collectors asked the mothers what immunizations the child had received up to and including 6-months (plus Vitamin A) and confirmed these answers with the child’s RtHC.

#### Child illnesses

Mothers were asked at the 6-months assessment if the child had experienced any child illness in the past 2 weeks including diarrhea, vomiting, cough, shortness of breath, fever, and/or runny nose. Mothers were asked if the mother has taken the child to the hospital, clinic, traditional healer, private doctor, or private pharmacy in the past 3 months.

#### Child development

WHO gross motor developmental milestones were measured at 6-months. The presence or absence of the following motor skills were assessed: (1) sitting without support; (2) standing with support; (3) hands-and-knees crawling; (4) walking with assistance; and (5) standing alone [32]. Depending on the child’s age (age unit: months), a child was determined to be either on target or behind target based on the assessment of the trained interviewer.

### PMTCT Cascade

Among the mothers living with HIV (MLH), the PMTCT cascade focused on six tasks: (1) receipt of antiretroviral (ARVs) before the birth of the baby; (2) nevirapine (NVP) administered to the infant after delivery; (3) continuation of Anti-retroviral Therapy (ART) for mother post-delivery; (4) infant received HIV PCR testing; (5) maintenance of a single feeding method (i.e. exclusive breastfeeding for 3 months); and (6) the infant received co-trimoxazole by the 6-months assessment.

### Data analysis

The key baseline demographics and maternal characteristics were compared between intervention and comparison groups, using the Student’s *t*-test or Mann-Whitney U test for continuous variables, and the chi-square (*χ*^2^) test or Fisher’s exact test for categorical variables. We also examined participants who were lost to follow-up (11%; *n* = 147), by comparing their baseline characteristics to mothers who were retained at 6-months assessment (*n* = 1142/1310) stratified by intervention and comparison groups.

Our primary analysis compared the distribution of maternal and child outcomes by intervention and comparison groups at 6-months using *t*-test and *χ*^2^ test for continuous and categorical outcomes, respectively. Among MLH, we compared additional tasks in the PMTCT cascade between intervention and comparison groups using *χ*^2^ test.

Longitudinal mixed-effects models were used to assess the intervention effect for maternal and child outcomes that were measured over time. In particular, we applied linear mixed-effects regression models with restricted maximum likelihood estimation for continuous outcomes, and mixed-effect logistic regression models for binary outcomes. The mixed-effects models for maternal and child outcomes assessed over time, included fixed-effects for maternal HIV status, access to electricity and safe water, having total grant income above 2000 South African Rand (ZAR), intervention, time, and interaction between intervention and time. The time variable used in the longitudinal models for maternal outcomes corresponds to time-points (baseline and 6-months) where mothers were assessed in the study, and for child outcomes refer to the actual age of children (age unit: months).

For child outcomes assessed only at 6-months, we omitted the child random-effects. Multivariate negative binomial regression models were fitted for count outcomes, where the likelihood ratio test was adapted to assess whether the negative binomial model was more appropriate than the Poisson model. For child outcomes that were assessed over time from baseline to 6-months, the effect modification was assessed by fitting interaction terms between maternal HIV status, time, and intervention in the longitudinal models. Similarly, for those child outcomes that were only measured at 6-months, this was assessed by including an interaction term between maternal HIV status and intervention in models. Further examination was carried out to assess whether maternal HIV status modifies the effect of intervention on outcomes.

All mixed-effects models accounted for repeated measures for participants by including random intercepts. Random slope for time was also assessed, and if needed, included in the models in addition to fixed-effects and random intercepts. All analyses were conducted using Stata SE software Version 15.

## Results

### Baseline differences

Follow-up assessments were completed by 89% of mothers and those lost to follow-up (*n* = 147) were similar to those retained (*n* = 1142) (details available from authors). Table 1 summarizes the background characteristics grouped by intervention condition. The median age of mothers was 24 years (Range, 14–50 years), with schooling for 8.8 years (SD, 2.6). About 38% of mothers (*n* = 493) were married or lived with a partner (*p* ≤ 0.05); unemployment rates were extremely high (72.1%) (*p* = 0.02). Mothers in the intervention group had lower total grant income (*p* < 0.01), and on average, had more antenatal visits (*p* < 0.01) and more previous pregnancies (*p* < 0.01) than those in the comparison group.

**Table 1:** Baseline characteristics of the sample by Intervention (*n* = 636) and comparison (*n* = 674) groups

	Intervention (n = 636)		Comparison (n = 674)		Total (***n*** = 1310)	
	n	(%)	n	(%)	n	(%)
*Demographic characteristics*						
Maternal age, median [range]	24 [14, 46]		23 [14, 50]		24 [14, 50]	
Highest education level, mean (SD)	8.7 (2.9)		8.9 (2.4)		8.8 (2.6)	
Employment^**^						
Employed	48 (7.6)		32 (4.8)		80 (6.1)	
School	123 (19.4)		161 (24.0)		284 (21.8)	
Unemployed	464 (73.1)		478 (71.2)		942 (72.1)	
Married or lives with partner^*^	257 (40.4)		236 (35.1)		493 (37.7)	
Total grant income (> 2000 Rand)^***^	177 (28.6)		247 (37.9)		424 (33.4)	
Total child support grant	577 (92.1)		588 (93.3)		1145 (92.7)	
Access to electricity^***^	272 (42.8)		553 (82.1)		825 (63.0)	
Access to safe water^***a^	607 (95.4)		264 (39.2)		871 (66.5)	
Number of adults in household, mean (SD)	3.4 (1.9)		3.6 (2.0)		3.5 (2.0)	
Number of children in household, mean (SD)	4.0 (1.9)		4.1 (1.9)		4.1 (1.9)	
*Alcohol*						
Use alcohol during pregnancy	48 (7.6)		42 (6.2)		90 (6.9)	
Use alcohol before pregnancy	36 (5.7)		46 (6.8)		82 (6.3)	
Continued to use alcohol after pregnancy discovery	2 (18.2)		6 (37.5)		8 (29.6)	
*Maternal and Mental health*						
Antenatal care	635 (99.8)		672 (99.7)		1307 (99.8)	
Total antenatal visits, mean (SD)^***^	4.3 (1.6)		3.9 (1.7)		4.1 (1.7)	
Total previous pregnancies*,* mean (SD) ^***^	1.5 (1.6)		1.3 (1.5)		1.4 (1.6)	
Number of live births, mean (SD)	2.1 (1.4)		2.0 (1.4)		2.1 (1.4)	
EPDS, median [range]	5 [0–27]		5 [0–30]		5 [0–30]	
EPDS > 13	79 (12.4)		86 (12.8)		165 (12.6)	
EPDS ≥18	26 (4.1)		31 (4.6)		57 (4.4)	
*HIV and Reproductive Health Behavior*						
Tested for HIV during pregnancy	568 (89.6)		604 (89.9)		1172 (89.5)	
Mothers living with HIV	187 (29.6)		204 (30.4)		391 (30.0)	
Ever receipt ARVs	183 (97.9)		202 (99.5)		385 (98.7)	
ARVs before pregnancy	90 (48.1)		86 (42.4)		176 (45.1)	
ARVs currently (Single pink pill-FDC or TEE)	172 (94.0)		194 (96.5)		366 (95.3)	
Disclosed HIV status	173 (92.5)		184 (90.6)		357 (91.5)	
Husband/boyfriend/partner	28 (16.2)		26 (14.1)		54 (15.1)	
Family (Mother/Sister/Mother-in-law, Other)	137 (79.2)		141 (76.6)		278 (77.9)	
Other (Friend/Mentor-mother/CHW/Neighbor)	8 (4.6)		17 (9.2)		25 (7.0)	
Number of people disclosed to, mean (SD)	1.7 (0.8)		1.8 (0.9)		1.8 (0.8)	
*Paternal characteristics*						
Child lives with father	108 (17.0)		98 (14.5)		206 (15.7)	
Father alcohol use	319 (51.9)		346 (54.7)		665 (53.3)	
Father tobacco use	188 (29.8)		197 (29.8)		385 (29.8)	
Father drug use	32 (5.2)		25 (3.9)		57 (4.5)	
Father living with HIV+	57 (30.5)		58 (28.6)		115 (29.5)	
*Breastfeeding*						
Breastfeeding at baseline interview	601 (94.5)		644 (95.6)		1245 (95.0)	
Breastfeed within 1 h of birth	546 (85.8)		556 (82.5)		1102 (84.1)	
*Child outcomes at birth*						
Birth in hospital ^*** b^	591 (92.9)		649 (96.3)		1240 (94.7)	
LBW (less than 2500 g)	65 (10.5)		73 (11.2)		138 (10.9)	
WAZ, mean (SD)	−0.6 (1.1)		−0.6 (1.1)		−0.6 (1.1)	
Underweight (WAZ < −2 SD)	57 (9.2)		63 (9.7)		120 (9.5)	
HAZ, mean (SD)	0.2 (1.5)		0.2 (1.5)		0.2 (1.5)	
Stunted (HAZ < −2 SD)	41 (6.5)		48 (7.2)		89 (6.8)	
WHZ, mean (SD)	−1.8 (2.3)		−1.6 (2.2)		−1.7 (2.2)	
Wasting (WHZ < -2 SD)	220 (37.2)		213 (33.5)		433 (35.3)	
Apgar Score at 5 min, mean (SD)	10.0 (0.7)		9.9 (0.8)		10.0 (0.8)	
Apgar Score at 5 min (7 and above)	588 (99.5)		641 (98.8)		1229 (99.1)	

^*^ *p* < 0.1; ^**^p < 0.05; ^***^p < 0.01

^a^ Safe water includes rainwater tank, communal tap running water, communal tap hand pump, hospital tap, running water tap on land, and non-safe water consists of river and well; ^b^ Hospital/clinic or on the way to hospital vs. Home

Abbreviations: *EPDS* Edinburgh Postnatal Depression Scale (EPDS). *LBW* Low birth weight (LBW); *WAZ* Weight-for-age z-score (WAZ). *HAZ* Length/height-for-age z-score (HAZ)

The two groups varied in the municipal services available in each area: mothers in the comparison group were about twice as likely to have electricity (*p* < 0.01) and not have safe water (*p* < 0.01) than mothers in the intervention group.

As shown in Table 1, about one-third of mothers reported living with HIV (*n* = 391; 30%), and just less than half (*n* = 176; 45.1%) were on HAART before the pregnancy. Almost all MLH received ART during pregnancy (98.7%), a majority of which were taking a single fixed dose combination (FDC) or Tenofovir/ Emtricitabine/Efavirenz (TEE) every day. MLH were much more likely to disclose their HIV status to family (*n* = 278; 77.9%) than to their partners (15.1%), but no differences were observed across the two groups and the average number of people disclosed to was two people (SD = 0.8).

Regarding parental characteristics, only 15.7% of mothers lived with the father of their child and about one-third of MLH reported that they knew that the HIV status of the father of their child was positive (29.5%); most mothers (53.3%) were not aware of the father’s HIV status.

There were no significant differences in mean WAZ, HAZ, and WHZ scores and the rate of low birth-weight infants, those stunted, underweight, and wasted across the two groups at birth, but the percentage of children wasted (WHZ < − 2) at birth was very high at 35%.

### Maternal outcomes at 6-months

Table 2 compares mothers in the intervention and comparison groups on EPDS outcomes at 6-months. While findings from the adjusted analyses highlighted higher EPDS scores for mothers in the comparison group compared to the intervention group (mean difference (MD): -0.9; 95% CI: − 1.7, − 0.2), there were no differences in the prevalence of having a depressed mood (EPDS > 13) or probable clinical depression (EPDS ≥18). No other significant differences on maternal outcomes between the intervention and control conditions were found.

**Table 2:** Maternal outcomes at 6-months grouped by intervention and comparison groups

	Intervention (***n*** = 565)		Comparison (***n*** = 577)		Estimated Mean Difference Intervention vs. Comparison	
Depression	Mean	SD	Mean	SD	Difference^a^	95% CI
EPDS score^**^	4.5	5.0	5.3	5.4	−0.9^c,d,††^	(−1.7, − 0.2)
Median [range] ^**^	3 [0–33]		4 [0–26]			
					**Estimated Odds Ratio** **Intervention vs. Comparison**	
**Depression**	n	%	n	%	OR^b^	95% CI
EPDS score > 13^**^	30	6.3	47	9.9	0.5^c,e^	(0.3, 1.1)
EPDS score ≥ 18	14	2.9	18	3.8	0.6 ^c,e^	(0.2, 1.8)

Multivariate models controlled for: maternal HIV status, access to electricity and safe water, and grant income above 2000 ZAR

^*^ *p*-value < 0.1,^, **^ p-value < 0.05, (*t*-tests or χ^2^ tests).^†^ p-value < 0.1, ^††^ *p*-value < 0.05, for regression analyses

^a^ Mixed-effects linear regression for continuous outcomes. ^b^ Mixed-effects logistic regression for binary outcomes

^c^ Random-intercept for mother

^d^ Random-slope for time using unstructured covariance

^e^ Random-slope for time using identity covariance structure

### Child outcomes at 6-months

As Table 3 shows, no significant differences were observed between the intervention and comparison groups in the adjusted analysis of the growth measures of HAZ and WAZ. However, the intervention group had higher mean for WHZ (MD: 0.3; 95% CI: 0.1, 0.6), and lower odds of wasting (WHZ < -2SD) (OR: 0.5; 95% CI: 0.3, 0.9) than the comparison group, as well as a trend toward a lower odds for being underweight (WAZ < − 2) (OR: 0.4; 95% CI: 0.1, 1.0; *p* < 0.01).

**Table 3:** Child outcomes at 6-months grouped by intervention and comparison groups

	Intervention (n = 565)		Comparison (***n*** = 574)		Estimated Mean Difference Intervention vs. Comparison	
	Mean	SD	Mean	SD	Difference^a^	95% CI
**Growth measures**						
Height-for-age Z-score (HAZ)^**^	0.5	1.6	0.7	1.6	−0.2^e,f^	(−0.4, 0.1)
Weight-for-age Z score (WAZ)	0.4	1.3	0.3	1.2	0.1 ^e,f^	(−0.1, 0.3)
Weight-for-height Z-score (WHZ) ^***^	0.3	1.5	−0.0	1.6	0.3^e,f †††^	(0.1–0.6)
					**Estimated Odds Ratio** **Intervention vs. Comparison**	
	n	%	n	%	OR^b^	95% CI
**Growth measures**						
Stunted (HAZ < -2 SD)	33	5.9	23	4.1	1.6^e,f^	(0.6, 3.9)
Underweight (WAZ < −2 SD)	13	2.3	22	3.9	0.4^e†^	(0.1, 1.0)
Wasting (WHZ < -2SD) ^***^	27	4.8	56	9.4	0.5^††^	(0.3, 0.9)
**Breastfeeding**						
Currently breastfeeding	274	48.5	301	52.4	0.5^e,g^	(0.1, 5.4)
					**Estimated Odds Ratio** **Intervention vs. Comparison**	
	n	%	n	%	OR^c^	95% CI
**Breastfeeding**						
Exclusive Breastfeeding for 3 months	349	61.8	352	61.3	1.6	(0.8, 3.3)
Exclusive Breastfeeding for 6 months	177	31.3	156	27.2	1.8^††^	(1.1, 2.9)
If not breastfeeding exclusively, started mixing formula with Nestum/Baby porridge^***^	380	80.3	447	88.3	0.4^††^	(0.2, 0.8)
Immunizations current^*^	302	57.5	280	52.0	1.1	(0.8, 1.6)
**Vitamin A has been given**	140	26.7	139	25.8	1.3	(0.8, 2.0)
**Child illness during the past 2 weeks**						
Diarrhoea	136	24.1	130	22.7	1.3	(0.9, 1.9)
Vomiting^**^	49	8.7	78	13.4	0.5^††^	(0.3,0.9)
Cough	250	44.3	279	48.6	0.6^††^	(0.4, 0.8)
Shortness of breath	95	16.8	115	20.0	0.8	(0.5, 1.2)
Fever	157	27.8	188	32.8	0.8	(0.6, 1.1)
Runny nose^**^	143	25.3	185	32.2	0.7^††^	(0.5, 0.9)
**Medical Visits (past 3 months)**						
Hospital visit^**^	24	4.3	43	7.5	0.5^†^	(0.3, 1.1)
Clinic for illness	300	53.1	313	54.5	0.8	(0.6, 1.2)
Consulted with traditional healer^***^	6	1.1	24	4.2	0.2^††^	(0.1, 0.8)
Consulted with private doctor^*^	29	5.1	46	8.0	0.6	(0.3, 1.2)
Consulted with a private pharmacy^***^	29	5.1	79	13.8	0.2^††^	(0.1, 0.4)
**WHO Development**						
On/Ahead target vs Behind target	399	93.0	415	91.2	1.3	(0.7, 2.6)
					**Estimated Incident Rate Ratio** **Intervention vs. Comparison**	
	n	%	n	%	IRR^d^	95% CI
**Total Count of Symptoms during the past 2 weeks**						
0	190	33.6	173	30.1	0.8^††^	(0.7,0.9)
1	137	24.3	128	22.3		
2	106	18.8	104	18.1		
3	74	13.1	81	14.1		
4	34	6.0	53	9.2		
5	21	3.7	26	4.5		
6	3	0.5	9	1.6		

Multivariate regression models controlled for: maternal HIV status, access to electricity and safe water, and having total grant income above 2000 ZAR

^*^ p-value < 0.1,^, **^ p-value < 0.05, ^***^ p-value < 0.001 (*t*-tests or χ^b^ tests); ^†^ p-value < 0.1, ^††^ p-value < 0.05, ^†††^ p-value < 0.001 for regression analyses

^a^ Mixed-effects linear regression for continuous outcomes; ^b^ Mixed-effects logistic regression for binary outcomes; ^c^ Multivariate logistic regression for binary outcomes; ^d^ Multivariate negative binomial regression for count outcomes

^e^ Random-intercept for child

^f^ Random-slope for time using unstructured covariance

^g^ Random-slope for time using identity covariance structure

Intervention mothers had higher odds of exclusive breastfeeding for 6 months (OR: 1.8; 95% CI: 1.1, 2.9) and lower odds of mixing formula with baby porridge (OR: 0.4; 95% CI: 0.2, 0.8). In addition, the odds of experiencing vomiting, cough and a runny nose in children of the intervention group were lower compared to children in the comparison group. The incident rate ratio for symptoms of common childhood illnesses was also lower for the intervention group (IRR: 0.8; 95% CI: 0.7, 0.9; *p* < 0.05).

Children in the intervention group had significantly lower odds of consulting with a traditional healer (OR: 0.2; 95% CI: 0.1, 0.8) and a private pharmacy (OR: 0.2; 95% CI: 0.1, 0.4) than children in the comparison group. No significant differences were observed between the two groups in terms of having up-to-date immunizations, receiving vitamin A, WHO developmental milestones, clinic visits for illness, or consulting with a private doctor in the past 3 months, but there was a trend towards fewer hospital visits for illness in the intervention group (OR: 0.5; 95% CI 0.3, 1.1).

Maternal HIV status tended to modify the effect of the intervention on exclusive breastfeeding for 6 months (*p* = 0.09; likelihood ratio test). In particular, the effect of the intervention was strong for mothers who were not HIV infected (OR: 2.1; 95% CI: 1.2, 3.4), indicating about a two-fold increase in the odds of exclusive breastfeeding compared to the non-MLH in the comparison group (see Table 4). There was no intervention effect observed on exclusive breastfeeding at 6 months for MLH. Maternal HIV status did not modify the effect of the intervention on any of the other maternal or child outcomes.

**Table 4:** Effect modification by maternal HIV status for exclusively breastfeeding for 6 months

Exclusively breastfeeding for 6 months	MLH		Non-MLH	
	OR	95% CI	OR	95% CI
Intervention vs. comparison group	0.9	(0.4, 2.3)	2.1	(1.2, 3.4)

### Interventions to protect HIV-exposed infants

**Fig. 3:**
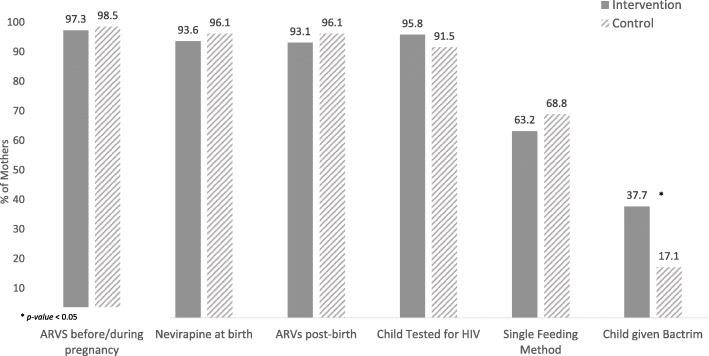
Interventions to protect HIV-exposed infants

MLH completed PMTCT tasks and other interventions to protect their HIV-exposed infants (Fig. 3) at similar rates with high compliance in terms of receiving ARVs before or during pregnancy (*n* = 382; 98.0%); administering infants Nevirapine (NVP) syrup soon after birth (*n* = 370; 94.9%); adhering to ARVs at 6-months (*n* = 282; 94.6%) and completing infant PCR testing (*n* = 278; 93.6%). However, MLH in the intervention group were much more likely to be giving infants co-trimoxazole at 6-months (37.7% vs 17.1%; *p* < 0.01) compared to MLH in the comparison group.

## Discussion

Home-visits by Philani CHWs had limited, yet important, health effects on mothers and their children. Maternal depressive symptoms were fewer and child health care behaviours were enhanced. Antenatal visits were higher, and exclusive breastfeeding rates at 6-months were higher (especially amongst HIV negative women) and mothers were less likely take their children to a traditional healer. MLH in the intervention group were more likely to be giving their children co-trimoxazole at 6-months. Children tended to have fewer hospital visits, fewer symptoms of illness in the preceding two weeks, were marginally less likely to be underweight-for-age and were significantly less likely to be wasted in the intervention group than the comparison group.

Many of these impacts can be attributed to specific interventions by CHWs: higher antenatal care attendance, higher exclusive breastfeeding rates and lower traditional healer visits are likely to be linked to strong repeated messages given by CHW during home visits. Other effects may be more indirect – lower average depression scores may be due to the benefits of peer support, while nutritional and health benefits may be due to better breastfeeding practices by women who were regularly visited and supported in this by CHW.

Mothers in the intervention condition had lower average depressed mood scores, but the clinical impact of this is difficult to quantify, as mean EPDS scores were well below 13. Most studies of maternal depression (even those using a screening tool) find rates of at least 20% [33, 34]. The impact of CHW on depression is consistent with previous findings in both peri-urban [35, 36] and rural settings (L Stansert Katzen et al. 2019 – in submission). This may be attributed to the emotional and practical support provided by CHW during home-visits and this is an important area for future research. Additionally, it is notable that this study is the second from the Eastern Cape where overall EPDS scores are much lower when compared to urban settings [37]. The lower levels of depression may be attributed to stronger family ties and community resources, despite poverty and inadequate government services, and a healthier living environment as compared to peri-urban settlements. More research is required to elucidate this.

Home visiting resulted in fewer underweight infants as well as wasted infants over time. Other studies have shown that maternal characteristics such as mother’s antenatal care and place of delivery were predictive of underweight, stunted and wasted infants. However, these data were cross-sectional and not longitudinal [20].

As expected, our findings of wasting is similar to a meta-analyses that found wasting is more prevalent in rural areas than compared to urban settings [38] and are more prevalent in households with low SES compared to higher SES [39]. However, the percentage of children who are wasted at birth (35%) is striking, even as this improves to 4.6% (intervention) and 9.4% (control) by 6 months. Additionally, mothers in the intervention group attended significantly more antenatal visits during pregnancy, which is linked to better pregnancy outcomes [40] and in line with WHO Antenatal Guidelines that promote additional antenatal visits. Intervention group mothers also engaged in healthier infant feeding behaviours. The appropriate feeding of infants is seen as critical to their health, growth and well-being [41], and mothers who received the intervention were more likely to exclusively breastfeed their infants for 6 months, and were less likely to mix cereal porridge into their formula bottle if not exclusively breastfeeding - which is regarded as a potentially harmful feeding practice [42]. A key aspect of the support CHWs provide is feeding advice – and it was important that there were higher levels of exclusive breastfeeding at 6 months and less mixing of porridge with formula. Breastfeeding rates were relatively high at about 61.6% (five times higher than in peri-urban settings [43].

Finally, in terms of clinical symptoms and care behaviours, infants in the intervention group were significantly less likely to have experienced one of six symptoms of common childhood illnesses, and independently had less vomiting, coughing and coryza (runny nose) than children in the comparison group. This may be due to the health benefits of higher exclusive breastfeeding rates in the intervention group or other care behaviours imparted by CHW to mothers. Hospital visits, a proxy for potentially serious illnesses, were also marginally less common in the intervention group. (Most women take their children to their nearest PHC clinic when unwell, children who are very ill or do not respond to clinic treatment are referred to the district hospital. On occasion, severely ill children will be taken directly to the hospital). Mothers in the comparison group were more likely to take their children to traditional healers and to private pharmacies for health consultations. The latter is most likely due to the fact that the comparison area is significantly closer to the nearest big town which has a private pharmacy with a consulting nurse on the premises. Consulting traditional healers is common in rural villages of South Africa, and may result in children being exposed to harmful or even fatal treatments [44, 45]. It is interesting that mothers in the comparison group were nearly five times the odds more likely to seek a traditional healer’s advice or treatment than those in the intervention group. This may be attributed to CHWs themselves having trust in local government PHC clinics and therefore facilitating access to clinics and the district hospital for mothers in the intervention group by providing referral letters or accompanying mothers to facilities.

The effect modification of HIV on exclusive breastfeeding at 6 months was notable: removing MLH from the analysis shifted the odds ratio of this practice from 1.8 to 2.1 for the intervention group (Table 4). We suspect that this is due to the strong ART programme at Zithulele Hospital and its clinics, where pregnant mothers are carefully counselled and encouraged to exclusively breastfeed, thereby increasing the proportion of mothers in the control area who are well counselled and therefore choose to exclusively breastfeed, diluting the measurable impact of the CHW breastfeeding counselling and support in the intervention area.

For MLH, the rate of co-trimoxazole uptake from 6 weeks after birth was significantly higher in the intervention group than in the comparison group, yet, at 37%, was still concerningly low. This may have been due to co-trimoxazole syrup stock-outs in clinics at the time of the study or nurses may have been unaware of co-trimoxazole guidelines for HIV-exposed infants. It is however encouraging that compliance of other interventions to protect HIV-exposed infants (see Fig. 3) in both conditions was higher than 90% - even in this deeply rural area - likely an important factor undergirding the dramatic decrease in HIV incidence in children in South Africa over the past years [46].

The Cape Town randomized controlled trial evaluating the CHW program showed more substantial impacts on mothers and their babies, particularly in “Interventions to protect HIV-exposed infants”, there referred to as the “PMTCT cascade” [18]. However, since the study in Cape Town was performed in 2008, the implementation of government PMTCT protocols have improved markedly in PHC clinics to close to 90% as illustrated above. The impact of CHW is therefore more difficult to quantify with baseline compliance rates so high.

### Limitations

While the matching of the two arms was mostly successful, there were significant differences between the two areas in terms of access to safe drinking water and electricity. The intervention area had good access to safe municipal water, but poor access to electricity, while in the comparison area the inverse was true. It is difficult to know what the impact of this balance of municipal services was on the mothers and their babies and whether one or other area was “advantaged” by this difference, although these were corrected for in the analyses. A cluster randomized controlled trial would be more robust to avoid confounders in further evaluations. We also did not assess the intervention dosage or have access to maternal HIV testing results and had to rely on self-report.

## Conclusion

This study shows limited but important effects of a CHW home-visiting program in a deeply rural area of South Africa. It appears that the positive impact of CHWs on health outcomes is more difficult to achieve in the rural context than in more densely populated urban and peri-urban areas [18]. This needs to be considered when implementing rural CHW programs and greater investments in transport and support of CHWs may be required to ensure the effectiveness of the intervention. It is important to recognise that the CHWs assessed in this study had only been established in the area for 12–18 months and that assessments of other CHW programs occurred after these programs had embedded themselves into communities over many years [18]. Nevertheless, mothers who were visited by CHWs before and for up to a 6 months after delivery exclusively breastfed for longer, attended antenatal care more often, had lower levels of depressive symptoms and higher trust in government healthcare services. Their children were also significantly less likely be wasted at 6-months. In conclusion, CHWs have an important role to play in the provision of PHC services in rural areas, but need to be managed in the light of the rural context and supported accordingly.

## Data Availability

The raw datasets generated and/or analyzed during the current study are not publicly available due to the sensitivity and availability of geographic data, but anonymized data sets are available from the corresponding author on reasonable request.

## Abbreviations

CHW: Community health workers
PHC: Primary healthcare clinics
SA: South African
NDOH: National Department of Health
NGO: Non-Governmental Organization
PMTCT: Prevention of mother-to-child HIV transmission
ZAR: South African Rand
EPDS: Edinburgh Postnatal Depression Scale
RtHC: Road-to-Health-Card
W.H.O: World Health Organization
HAZ: Height-for-age z scores
WAZ: Weight-for-age z scores
WHZ: Weight-for-height z scores
MLH: Mothers living with HIV
ARV: Antiretroviral
NVP: Nevirapine
ART: Anti-retroviral therapy
FDC: Fixed dose combination
TEE: Tenofovir/ Emtricitabine/Efavirenz
